# Lipoxygenase-derived oxylipins are enriched in anti-citrullinated protein antibody (ACPA)-positive individuals at risk for developing rheumatoid arthritis

**DOI:** 10.1186/s13075-024-03274-0

**Published:** 2024-02-15

**Authors:** Liam J. O’Neil, Vidyanand Anaparti, Tanja Winter, Irene Smolik, Xiaobo Meng, Harold M. Aukema, Hani El-Gabalawy

**Affiliations:** 1 Department of Internal Medicine, Rady Faculty of Health Sciences, University of Manitoba, Winnipeg, Canada; 2 Manitoba Centre for Proteomics and Systems Biology, University of Manitoba, Winnipeg, Canada; 3 Department of Food and Human Nutritional Sciences, University of Manitoba, Winnipeg, Canada; 4 Canadian Centre for Agri-Food Research in Health and Medicine, St. Boniface Hospital Albrechtsen Research Centre, Winnipeg, Canada

**Keywords:** Rheumatoid arthritis, ω-6 fatty acids, ω-3 fatty acids, Oxylipins, Preclinical RA, Polyunsaturated fatty acids, PUFA, Fatty acids

## Abstract

**Background:**

Rheumatoid arthritis (RA) is typically preceded by an extended preclinical period where circulating autoantibodies, particularly anti-citrullinated protein antibodies (ACPA), are detectable in the absence of clinical arthritis. Increased dietary intake of anti-inflammatory omega-3 (ω3) polyunsaturated fatty acids (PUFA) has been shown to be associated with a lower the risk of developing incident RA in large epidemiological studies. It is currently not known how changes in fatty acid (FA) metabolism may impact on the progression towards RA in at-risk individuals. To begin to address this question, we profiled serum FAs and oxylipins in an established cohort of at-risk ACPA-positive first-degree relatives (FDR) of RA patients (*N* = 31), some of whom developed RA (*N* = 4), and compared their profile to ACPA-negative FDR from the same population (*N* = 10).

**Methods:**

Gas chromatography (GC) was used for FA quantitation. Oxylipins were extracted and quantified using high-performance liquid chromatography–tandem mass spectrometry (HPLC/MS/MS).

**Results:**

Although we did not detect any meaningful differences in overall FA content between ACPA + and ACPA − FDR, the levels of oxylipins derived from FA metabolism demonstrated significant differences between the two groups, with the ACPA + group demonstrating enrichment in circulating arachidonic acid- and eicosapentaenoic acid-derived molecules. Compared with the ACPA − FDR group, the ACPA + FDR, including those who progressed into inflammatory arthritis, displayed higher levels of LOX-derived oxylipins.

**Conclusion:**

ACPA seropositivity in otherwise unaffected individuals at-risk for developing future RA based on family history (FDR) is associated with alterations in the serum oxylipin profile that suggests dysregulated LOX activity.

**Supplementary Information:**

The online version contains supplementary material available at 10.1186/s13075-024-03274-0.

## Introduction

Rheumatoid arthritis (RA) is a systemic autoimmune disorder characterized by persistent inflammation in the synovium leading to progressive articular damage [1]. It is now well established from multiple retrospective and prospective cohorts that prior to the onset of arthritis, autoantibodies develop in the pre-clinical stage of the disease, most commonly anti-citrullinated protein antibodies (ACPA) and rheumatoid factor (RF) [2]. Although ACPA + individuals are at increased risk to develop incident RA, the pathogenic events that ultimately leads to the onset of arthritis remain incompletely understood [3].

Dietary fatty acids such as omega-3 (ω3) polyunsaturated fatty acids (PUFA) display anti-inflammatory properties primarily though the action of their oxidized metabolites, known as oxylipins [4]. While ω3 FA intake seems to be protective against the development of RA, the mechanisms by which this occurs have not been elucidated [5]. Oxylipin profiles in a small cohort of individuals with clinically suspect arthralgia (CSA), some of whom were ACPA + , resemble those in early treatment naïve RA and are most suggestive of activation of the lipoxygenase (LOX) pathway [6].

In order to better understand the potential role of fatty acids and their oxylipin metabolites in a individuals who are at increased risk of developing RA, we undertook an exploratory, targeted FA and oxylipin analysis of a cohort of ACPA + FDR of Indigenous North American RA patients, a population that we have shown to be at particularly high risk of developing future RA [2].

## Methods

### Study design

Study participants were recruited from multiple Cree, Ojibway, and Ojicree First Nation (FN) communities in Central Canada [2]. ACPA seropositivity was determined based on the results of commercially available anti-cyclic citrullinated peptide (CCP) assays (either CCP-2 [Euro Diagnostica] or CCP-3 [Inova] were used; with either assay, ACPA positivity was demonstrated at any level > 20 U/ml, based on the manufacturer’s instructions). Based on this testing, the following 2 groups were included in this study: ACPA + FDR (*N* = 53) and ACPA − FDR (*N* = 10), which served as the control group. Some of the FDR reported musculoskeletal pain in various locations, as we have documented in other publications [7]. Venous blood was collected into SST™ serum separation tubes (BD Vacutainer Systems) and centrifuged for serum separation as per the manufacturer’s instructions. Serum was stored at – 20 °C until further use. The analysis was conducted in 2018.

### Fatty acid analysis

Total lipids were extracted and quantified from 250 μL aliquots of serum according to our optimized methodology as published previously [8]. Briefly, solvent–solvent extraction method was used for extracting lipids. Following this, lipids were methylated and quantified by gas–liquid chromatography. Values were expressed as micrograms per milliliter of serum and classified as either polyunsaturated (PUFA), monounsaturated (MUFA), or saturated (SFA).

### Oxylipin analysis

Two hundred fifty microliters of serum samples were prepared for oxylipin analysis and quantified using a HPLC–MS-MS method by multiple reaction monitoring as described previously [9, 10]. The stable isotope dilution method was used to quantify oxylipin levels, which were represented as nanograms per microliter of serum. Of all the screened oxylipins, metabolites with values below the lower limit of quantitation were excluded, while oxylipins whose expression was identified in ~ 95% of the samples were used for subsequent analyses. Oxylipins were classified based on their derived molecule: arachidonic acid (AA), linoleic acid (LA), dihomo-y-linolenic acid (DGLA), alpha-linolenic acid (ALA), eicosapentaenoic acid (EPA), and docosahexaenoic acid (DHA).

### Data analysis and statistics

Considering the samples used in this study were collected over a 15-year time span, we analyzed the effect of sample storage time on total FA and oxylipin levels. While we did not observe any effect of sample storage time on the mean total FA concentrations in samples collected between 2006 and 2017, most PUFA levels were lower in the “before 2013” group compared with “after 2013” (Table S1 and Figure S1A). Mean serum concentrations of total oxylipins were significantly higher in samples collected and stored before 2013 (*n* = 22) compared with samples collected after 2013, and this correlated significantly with storage time (Figure S1B, *P* < 0.0002). Based on these data, we used FA and oxylipin levels only from samples collected *after* 2013 (*n* = 31) for the remaining analyses. As a secondary analysis, we used linear regression to adjust oxylipin levels for sample storage time in samples collected after 2013. We also observed that NSAID use did not affect the levels of both FAs and oxylipins (Figure S1C, D). Therefore, we did not correct for any NSAID use in our study samples. MetaboAnalyst 5.0 (www.metaboanalyst.ca), GraphPad Prism (version 8.0), and R were used for data analysis, and data was log transformed. Kruskal–Wallis test with Dunn’s post hoc method, Pearson’s chi-square test, Mann–Whitney *U* test, Spearman’s rank correlation analyses, and logistic regression were used for statistical analysis as required, and *P-*values < 0.05 were considered as significant. Benjamini–Hochberg method was used to correct for false discovery rate (FDR). Unsupervised k-means clustering was undertaken using the R package *ConsensusClusterplus.*

## Results

### FA differences in ACPA positive and negative FDR

Baseline characteristics of FDR are shown in the Table S2, split by ACPA status. Approximately 75% of the study participants were female with a mean age of 46.8 ± 13.6 (mean ± SD) years. Of note, ACPA + FDR reported a higher frequency of NSAID use compared with ACPA − FDR (*P* = 0.004). BMI (30 vs 24.8, *P* = 0.02) and age (47.0 vs 27.1, *P* < 0.0001) were also higher in ACPA + FDR. ACPA + samples included participants with prevalent ACPA positivity (selected sample positive at first visit, *n* = 10), a longitudinal sample from an individual who was persistently ACPA + (*n* = 9) or incident ACPA (an individual who was ACPA − , but subsequently became ACPA + , *n* = 12, Table S3).

**Fig. 1:**
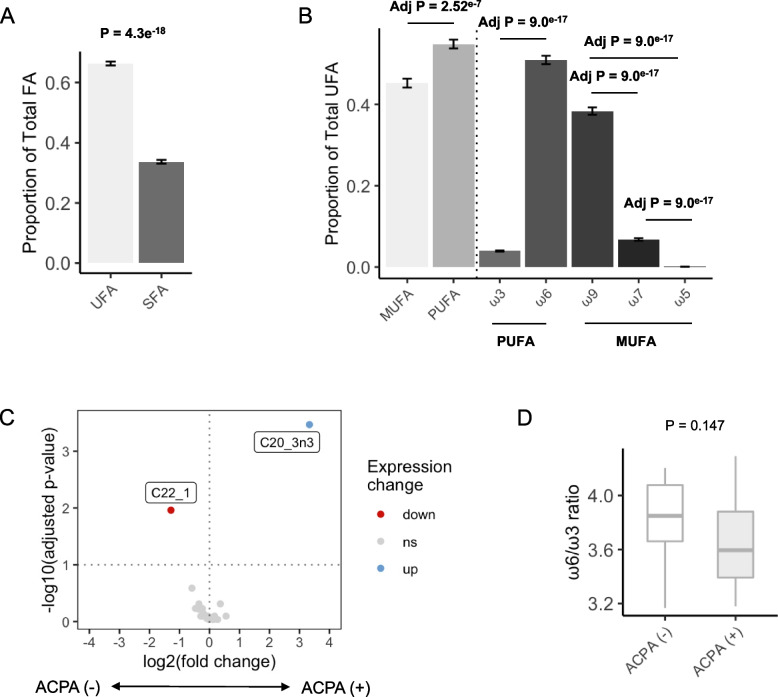
Distribution of fatty acids and differences in ACPA + and ACPA − FDR. The samples used for this analysis were collected in/after 2013. **A** Bar plots showing relative proportion of unsaturated (UFA) and saturated (SFA) fatty acids in all samples. **B** Bar plot showing relative proportions of mono-unsaturated (MUFA), polyunsaturated (PUFA), and omega-3, omega-6, omega-9, and omega-5 fatty acids. **C** Volcano plot of differentially expressed FAs between ACPA + and ACPA − FDR. Analyzed by pairwise Wilcoxon rank sum test and adjusted for multiple comparisons. Significant FAs are annotated on the graph (erucic acid = C22_1, eicosatetraenoic acid = C20_3n3). **D** The ratio between omega-6 (ω6) and omega-3 (ω3) FA in ACPA − and ACPA + FDR. Differences determined with Wilcoxon rank-sum test

Overall, 28 FAs were detected in the samples, of which unsaturated fatty acids (UFA) were significantly overrepresented compared with SFA (66.3% vs 33.7%, *P* < 0.00001, Fig. 1A). Among the UFA, 45.2% were MUFA (38.3% ω9, 6.8% ω7 and 0.08% ω5) and 54.8% were PUFA (3.9% ω3 and 50.1% ω6, Fig. 1B). Group level differences were determined between ACPA + and ACPA − FDR which revealed higher levels of erucic acid, (C22:1, log2 fc =  − 1.2, *P* = 0.01) an ω6 FA, in ACPA − FDR (Fig. 1C). Conversely, eicosatrienoic acid (n3) (ETE, C20:3n3), an ω3 FA, was significantly higher in ACPA + FDR (log2 fc = 3.3, *P* = 0.003, Table S4, Fig. 1C). Consistent with this, the total ω6/ω3 ratio trended lower in ACPA + FDR compared with ACPA − FDR (14.4 vs 12.1, *P* = 0.15, Fig. 1D) although we did not observe any other individual FAs that trended towards significance. Indeed, the distribution of FAs (% PUFA, MUFA, SFA, UFA, or any of the PUFA/MUFA subclasses) displayed no differences or trends based on ACPA seropositivity (Figure S2). Overall, these data suggest FA profiles vary based on ETE (high) and erucic acid (low) in ACPA + FDR.

### Shared oxylipin profiles in ACPA + FDR and ACPA + progressors

**Fig. 2:**
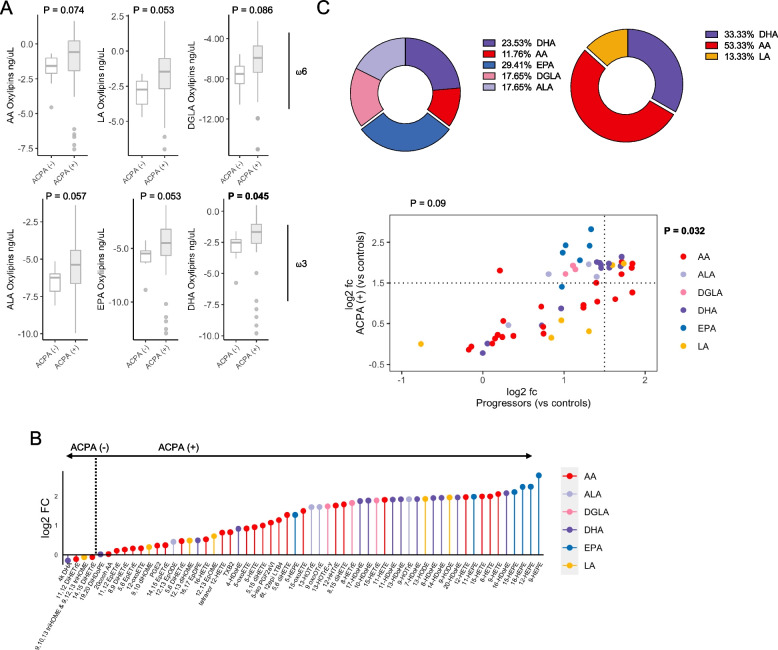
Differences in oxylipin profiles in various pre-clinical RA stages. (**A**) Oxylipins derived from arachidonic Acid (AA), linoleic Acid (LA), dihomo-y-linolenic acid (DGLA), alpha-linolenic acid (ALA), eicosapentaenoic Acid (EPA) and Docosahexaenoic acid (DHA) in ACPA- FDR and ACPA+ FDR. Differences determined with Wilcoxon rank-sum test. (**B**) Ordered oxylipins by log2 fold change (vs ACPA- FDR) colored by derived fatty acid (legend). (**C**) Log2 fold change in Progressors (vs ACPA- FDR) or ACPA+ FDR (vs ACPA- FDR) categorized by log2 fold change > 1.5 (dotted lines). Pie charts are representative of the proportion of oxylipins based on the FA from which they are derived. Differences in proportion measured with chi-square test. The ratio between AA-derived oxylipins and EPA-derived oxylipins in ACPA+ FDR and ACPA+ Progressors. Differences determined with Wilcoxon rank-sum test

Given the differences observed between ACPA + and ACPA − FDR in individual FA, we next sought to determine if levels of oxylipins also differed based on ACPA status. Trends were observed for increased oxylipins derived from both ω3 FA (ALA, EPA) and ω6 FA (AA, LA, DGLA, Fig. 2A) with differences in ω3 DHA oxylipins reaching statistical significance (*P* = 0.045, Figure S3 shows data adjusted for storage time). However, within-group differences were predominantly observed in ω6-derived oxylipins (Figure S4), rather than ω3-derived oxylipins. Unsupervised clustering of individual oxylipins revealed 8 distinct clusters, and the expression of oxylipins within 2 of these clusters was higher in ACPA + FDR compared with ACPA − (cluster 1 *p* = 0.037, cluster 5 *p* = 0.028, Figure S5). Analysis of individual oxylipins in ACPA + FDR showed that the levels of 4 oxylipins (4-HDoHE, 9-HODE, 13-HODE, 8,15 DiHETE, and 5-HETE) were significantly higher compared with ACPA − FDR (Table S5). We then ranked oxylipin expression based on log2 fc and observed that of the top differentially expressed oxylipins, the predominant representation (top 10) was from EPA-derived (50%) and AA-derived (40%) molecules (Fig. 2B). Taken together, these findings demonstrate an increased oxylipins in ACPA + FDR with a higher abundance in both ω3 and ω6 PUFA oxylipins.

Given the longitudinal nature of the cohort, we undertook an exploratory analysis to determine if oxylipin profiles differed based on whether or not ACPA + FDR eventually progressed into clinical RA (*n* = 4, Table S6). Oxylipin levels in ACPA + progressors displayed similar patterns with ACPA + FDR (non-progressors), with higher levels of total oxylipins and trend lines suggestive of increasing levels of AA and LA (ω6) oxylipins (Figure S3, Figure S6). Using ACPA − FDR as the reference group, we categorized individual oxylipins that were higher in ACPA + FDR, higher in ACPA + progressors, or shared with both groups. Interestingly, ACPA + FDR and progressors shared an enrichment of AA-derived ω6 and LA-derived ω6 oxylipins (53.3% and 13.3% respectively, *P* = 0.03), while the ACPA + FDR uniquely expressed higher levels of EPA-derived (29.4%, ω3), ALA-derived (17.7%, ω3), and DGLA-derived (17.7%, ω6) oxylipins (*P* = 0.09, Fig. 2C). Hence, although ACPA + FDR and progressors displayed clear similarities in their oxylipin expression profiles, there was a suggestion that subtle differences may be identifiable prior to the onset of arthritis.

### Activation of LOX is upstream to oxylipin alterations in ACPA + FDR

**Fig. 3:**
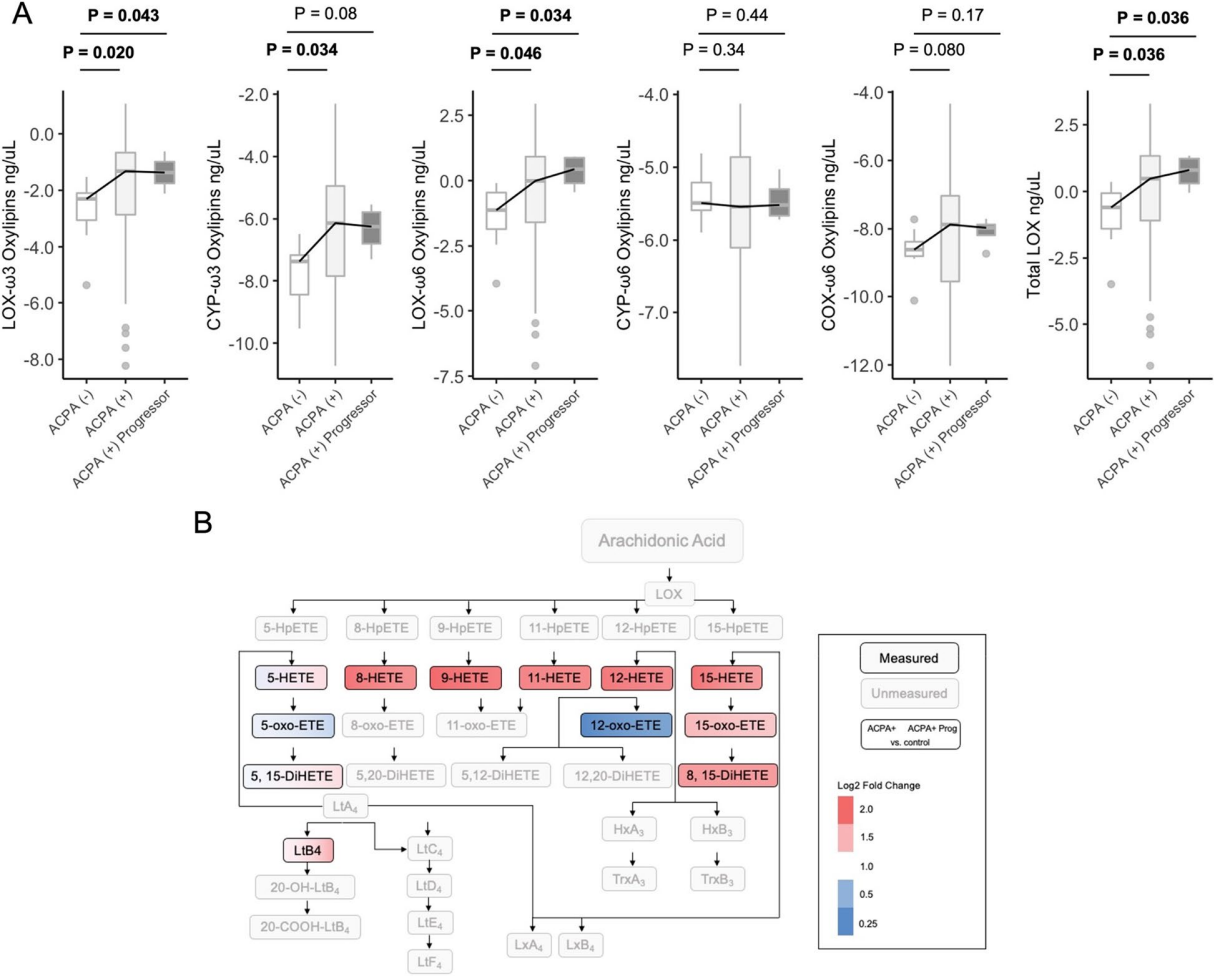
LOX-derived oxylipins are increased in ACPA+ FDR and ACPA+ Progressors. (**A**) Omega-6 (ω6) and omega-3 (ω3) oxylipins derived from LOX (lipoxygenase), COX (cyclooxygenase), or CYP (cytochrome P450) pathways in ACPA- FDR, ACPA+ FDR and ACPA+ Progressor. Differences determined with Wilcoxon rank-sum test. Trend lines are drawn through the median values for each group (**B**) Arachidonic acid lipoxygenase pathway annotated by known steps of synthesis to derive oxylipins. Undetected oxylipins are grey, while detected are colored in black. Within each cell, there is a color gradient from left to right, based on the expression relative in ACPA+ FDR (left) and ACPA+ Progressors (right) relative to controls (log2 fold change)

Given the finding that AA-derived oxylipins were enriched in ACPA + FDR, we next sought to delineate the enzymatic pathways that may be responsible for some of these differences. An analysis of upstream enzyme activity (LOX, COX, or CYP) displayed an over-expression LOX-ω3 (*P* = 0.020), CYP-ω3 (*P* = 0.034), and LOX-ω6 (*P* = 0.046) in ACPA + FDR with similar findings in LOX-ω3 (*P* = 0.043) and LOX-ω6 (*P* = 0.034) in ACPA + progressors (Fig. 3A, Figure S3). Indeed, total LOX-derived oxylipins were higher in ACPA + FDR (*P* = 0.036) and ACPA + progressors compared with ACPA − FDR (*P* = 0.036). Using logistic regression, we found that LOX-derived oxylipins were higher in ACPA + samples compared to ACPA − , after controlling for BMI and age (OR 11.9, 95% CI 1.5 to 147.5, *P* = 0.03). Furthermore, from our unsupervised clustering analysis, LOX-derived oxylipins were enriched in both cluster 1 (11/13) and cluster 5 (5/5), and ACPA status was associated with oxylipin expression for both clusters in a logistic regression model (cluster 1, OR = 14.6 95% CI 1.8 to 190.4, *P* = 0.02, cluster 5 OR = 28.2 95% CI 2.8 to 759.1, *P* = 0.01). Annotation of the AA-LOX pathway revealed several pathway members that were highly expressed in both ACPA + and ACPA + progressors compared with ACPA − FDR (Fig. 3C).

## Discussion

Although the role of dietary FAs and their downstream metabolites such as oxylipins have been described, little is known regarding how these molecules might contribute to the specific stages of pre-clinical RA. To address this, we performed an exploratory analysis of the circulating FA and oxylipin composition in a prospective cohort of ACPA + and ACPA − FDR. We demonstrate that ACPA + FDR display a higher abundance of oxylipins compared with ACPA − FDR, many of which were derived through upstream lipoxygenase activity.

The lipoxygenase family of non-heme containing lipid peroxidases generate pro- and anti-inflammatory mediators, with several key functions such as monocyte recruitment, regulation of phagocytosis, and establishing immune homeostasis [11]. Interestingly, these results are consistent with a recent study suggesting that LOX is activated in individuals with clinically suspect arthralgia, a precursor to RA [6]. Similarly, increased levels of 5-HETE were associated with progression into inflammatory arthritis in an at-risk cohort of ACPA + FDR [12]. Although we observed AA-derived oxylipins enriched in both ACPA + FDR and ACPA + progressors, enrichment in EPA-derived oxylipins was only found in ACPA + FDR. We speculate that ω3 oxylipins may be protective against the development of RA in ACPA + individuals, though reproducing this finding in a larger cohort will be essential.

Our study has a few limitations. Due to sample availability, circulating FA and oxylipin composition were determined using serum samples. While FA quantification in plasma or serum is reliable and clinically useful [13], FA in erythrocyte membranes is more reflective of long-term dietary intake, endogenous FA metabolism, and subsequent tissue assimilation [14]. Indeed, only subtle differences in FA content between ACPA + / − samples were observed, possibly due to sample type (serum), but this may also be related to samples that were obtained in a non-fasting state [15]. Due to the size of our cohort study, our biobank was limited to storage at – 20 °C, which likely influenced oxylipin profiles of samples stored before 2013. Furthermore, only a small number of ACPA + FDR were classifiable as progressors after longitudinal follow-up, which limits the power of comparing progressors from non-progressors. Finally, the samples analyzed were from one time point, and no inference could be made regarding the turnover of FA and oxylipins. Hence, the increase in oxylipins observed in ACPA + FDR could be due to increased synthesis, reduced degradation, or a combination of both.

## Conclusions

Overall, our exploratory study provides insights into FA and oxylipin metabolism in preclinical RA. Future investigation is required to advance our understanding the mechanistic relationships between autoantibodies and FA/oxylipin metabolism and their eventual contribution towards progression of RA from a pre-clinical state.

### Supplementary Information

**Additional file1:** **Table S1.** Effect of storage time on the levels of individual FAs – Columns highlighted in bold represent FAs that showed significant differential expression. Data was analyzed by student t test (assuming unequal variance) and *q*<0.05 was considered significant. Benjamini-Hochberg method was used to correct of false discovery rate (represented as *q*-values). **Table S2.** Clinical features of ACPA− and ACPA+ FDR. RF=rheumatoid factor; CRP = C-reactive protein; DAS28 = disease activity score 28; anti-CCP = anti-cyclic citrullinated protein antibodies; BMI = Body Mass Index. ^#^Pearson Chi-square test; ^$^Mann-Whitney U test; statistically significant values are indicated in bold. ‘-‘ indicates absence of any value. **Table S3.** Characteristics of ACPA+ samples selected for fatty acid and oxylipin analysis, categorized by either i. sample acquired at inception study visit or ii. sample acquired after longitudinal follow-up. **Table S4.** Table showing differences in FA levels between ACPA+ and ACPA− FDR. Data analyzed Mann-Whitney U test and false-discovery rate was corrected using Benjamini-Hochberg method. Significant values are indicated in bold. **Table S5.** Table showing differences in individual oxylipin levels between ACPA+ and ACPA− FDR. Data is represented as mean ± SD. *P* values were obtained after performing Student *t*-test and correcting for multiple comparisons using Bonferroni-Dunn method. Significant values were indicated in bold. **Table S6.** Characteristics of ACPA+ and ACPA+ Progressors. **Figure S1.** (A) Scatter plot showing the total FA levels quantified in samples segregated based on the year of sample collection and the Spearman rank correlation with years of storage. (B) Scatter plot showing the total oxylipins in samples segregated based on the year of sample collection and the Spearman rank correlation with years of storage. (C) Scatter plot showing the concentrations of total FA mass and total oxylipin mass in all individuals segregated based on (+/-) NSAID use. Data analyzed by Mann-Whitney U test. Samples used for this analysis were collected between 2007-2017. (D) Scatter plot showing the concentrations of total FA mass and total oxylipin mass in all individuals segregated based on enzymatic pathway and (+/-) NSAID use. Data analyzed by Mann-Whitney U test. Samples used for this analysis were collected after 2013. **Figure S2.** Analysis and distribution of FAs in FDR. Samples used for this analysis were collected in/after 2013. Box-Whiskers plots showing the % distribution of serum (A) MUFA and PUFA (B) SFA and UFA and (C) ω3, ω6, ω9, ω7 and ω5 FA subtypes in ACPA− FDR, and ACPA+ FDR. **** = *P *< 0.0001, ns = not significant; data analyzed by Mann-Whitney U test. **Figure S3.** Levels of serum oxylipins in ACPA+, ACPA− and ACPA+ Progressors after adjustment for sample storage time. **Figure S4.** Box-Whiskers plot showing levels of ω3 and ω6 oxylipins in ACPA− FDR (*N* = 10) and ACPA+ FDR (*n* = 31). ** = *P *< 0.01, ns = not significant. Data was analyzed using Mann-Whitney U test. **Figure S5.** Consensus clustering of oxylipins revealed 8 distinct clusters, 2 of which were higher in ACPA+ FDR samples compared with ACPA−. Analyzed by Wilcoxon rank sum test. MDS: Multi-dimensional scaling. **Figure S6.** Total Oxylipin levels in ACPA− FDR, ACPA+ FDR, and ACPA+ Progressors (top). Levels of AA-, LA-, DGLA-, ALA-, EPA-, and DHA-derived oxylipins split by group.

## Data Availability

Data is available upon request.

## Abbreviations

RA: Rheumatoid arthritis
ACPA: Anti-citrullinated protein antibodies
PUFA: Polyunsaturated fatty acids
FA: Fatty acids
FDR: First-degree relative
GC: Gas chromatography
HPLC: High-performance liquid chromatography
MS: Mass spectrometry
LOX: Lipoxygenase
RF: Rheumatoid factor
CSA: Clinically suspect arthralgia
FN: First Nations
CCP: Cyclic citrullinated peptide
MUFA: Mono unsaturated fatty acids
SFA: Saturated fatty acids
AA: Arachidonic acid
LA: Linoleic acid (LA)
DGLA: Dihomo-y-linolenic acid
ALA: Alpha-Linolenic acid
EPA: Eicosapentaenoic acid
DHA: Docosahexaenoic acid
NSAID: Non-steroidal anti-inflammatory
BMI: Body mass index
ETE: Eicosatrienoic acid
COX: Cyclooxygenase
CYP: Cytochrome P450
