# Supplementary material for: Lipoxygenase-derived oxylipins are enriched in anti-citrullinated protein antibody (ACPA)-positive individuals at risk for developing rheumatoid arthritis
Source: Arthritis Res Ther. 2024 Feb 15;26:51. doi: 10.1186/s13075-024-03274-0 (PMC10868017; doi:10.1186/s13075-024-03274-0)

**Table S1:** Effect of storage time on the levels of individual FAs – Columns highlighted in bold represent FAs that showed significant differential expression. Data was analyzed by student t test (assuming unequal variance) and *q*<0.05 was considered significant. Benjamini-Hochberg method was used to correct of false discovery rate (represented as *q*-values).

**Table S2:** Clinical features of ACPA- and ACPA+ FDR. RF=rheumatoid factor; CRP = C-reactive protein; DAS28 = disease activity score 28; anti-CCP = anti-cyclic citrullinated protein antibodies; BMI = Body Mass Index. ^#^Pearson Chi-square test; ^$^Mann-Whitney U test; statistically significant values are indicated in bold. ‘-‘ indicates absence of any value.

**Table S3:** Characteristics of ACPA+ samples selected for fatty acid and oxylipin analysis, categorized by either i. sample acquired at inception study visit or ii. sample acquired after longitudinal follow up.

| ACPA+ FDR (n = 31) | |
| --- | --- |
| **Inception Study Visit, n(%)** | 10 (32.3) |
| **Longitudinal, n(%)** | 21 (67.7) |
| Follow up, months, median (IQR) | 79 (25) |
| Time since 1st ACPA+, months, median (IQR) | 27 (79) |

**Table S4:** Table showing differences in FA levels between ACPA+ and ACPA- FDR. Data analyzed Mann-Whitney U test and false-discovery rate was corrected using Benjamini-Hochberg method. Significant values are indicated in bold.

| FA | p-value | adj.p-value | p.adj.signif | class | log2FC |
| --- | --- | --- | --- | --- | --- |
| C14_0 | 0.087 | 0.488 | ns | ns | 0.359 |
| C14_1 | 0.574 | 0.804 | ns | ns | 0.169 |
| C16_0 | 0.917 | 0.917 | ns | ns | -0.021 |
| C16_1n7cis | 0.693 | 0.844 | ns | ns | -0.084 |
| C16_1n7trans | 0.233 | 0.725 | ns | ns | -0.201 |
| C17_0 | 0.917 | 0.917 | ns | ns | 0.269 |
| C18_0 | 0.693 | 0.844 | ns | ns | 0.081 |
| C18_1 | 0.852 | 0.917 | ns | ns | 0.110 |
| C18_1n7c | 0.370 | 0.804 | ns | ns | -0.191 |
| C18_2n6 | 0.603 | 0.804 | ns | ns | -0.131 |
| C18_3n3 | 0.884 | 0.917 | ns | ns | 0.140 |
| C18_3n6 | 0.370 | 0.804 | ns | ns | -0.090 |
| C20_0 | 0.603 | 0.804 | ns | ns | -0.170 |
| C20_1 | 0.416 | 0.804 | ns | ns | -0.045 |
| C20_2n6 | 0.492 | 0.804 | ns | ns | -0.293 |
| **C20_3n3** | **< 0.001** | **< 0.001** | ******* | **up** | **3.331** |
| C20_3n6 | 0.441 | 0.804 | ns | ns | -0.185 |
| C20_4n6 | 0.519 | 0.804 | ns | ns | -0.229 |
| C20_5n3 | 0.603 | 0.804 | ns | ns | -0.131 |
| C22_0 | 0.348 | 0.804 | ns | ns | -0.236 |
| **C22_1** | **0.001** | **0.011** | ***** | **down** | **-1.285** |
| C22_2n6 | 0.492 | 0.804 | ns | ns | 0.551 |
| C22_4n6 | 0.028 | 0.259 | ns | ns | -0.588 |
| C22_5n3 | 0.135 | 0.588 | ns | ns | -0.470 |
| C22_5n6 | 0.173 | 0.606 | ns | ns | -0.359 |
| C22_6n3 | 0.755 | 0.881 | ns | ns | -0.092 |
| C24_0 | 0.072 | 0.488 | ns | ns | -0.352 |
| C24_1 | 0.147 | 0.588 | ns | ns | -0.241 |

**Table S5:** Table showing differences in individual oxylipin levels between ACPA+ and ACPA- FDR. Data is represented as mean + SD. *P* values were obtained after performing Student *t*-test and correcting for multiple comparisons using Bonferroni-Dunn method. Significant values were indicated in bold.

| **Oxylipin (ng/µL)** | **Precursor FA** | ***P-value (CCPneg vs CCPpos)*** | **CCPpos mean** | **CCPpos sd** | **CCPneg mean** | **CCPneg sd** |  |
| --- | --- | --- | --- | --- | --- | --- | --- |
|  |  |  |  |  |  |  |  |
| **ω-3 FA** | |  |  |  |  |  |  |
| **LOX Pathway** | |  |  |  |  |  |  |
| 9-HOTrE | ALA | 0.4347 | 0.021 | 0.034 | 0.006 | 0.004 |  |
| 13-HOTrE | ALA | 0.4628 | 0.022 | 0.035 | 0.007 | 0.004 |  |
| 9 oxoOTrE | ALA | 0.9576 | 0.001 | 0.003 | 0 | 0 |  |
| 9-HEPE | EPA | 0.7211 | 0.008 | 0.017 | 0.001 | 0.001 |  |
| 12-HEPE | EPA | 0.7812 | 0.007 | 0.012 | 0.001 | 0.001 |  |
| 15-HEPE | EPA | 0.7525 | 0.008 | 0.015 | 0.002 | 0.001 |  |
| 5-HEPE | EPA | 0.2113 | 0.041 | 0.039 | 0.016 | 0.009 |  |
| 11-HEPE | EPA | 0.8632 | 0.004 | 0.007 | 0.001 | 0.001 |  |
| 10-HDoHE | DHA | 0.7526 | 0.008 | 0.01 | 0.002 | 0.001 |  |
| 11-HDoHE | DHA | 0.7204 | 0.009 | 0.011 | 0.003 | 0.001 |  |
| 13-HDoHE | DHA | 0.5103 | 0.018 | 0.02 | 0.005 | 0.003 |  |
| 14-HDoHE | DHA | 0.5403 | 0.016 | 0.02 | 0.004 | 0.003 |  |
| 16-HDoHE | DHA | 0.5737 | 0.014 | 0.02 | 0.003 | 0.002 |  |
| 17-HDoHE | DHA | 0.1438 | 0.041 | 0.054 | 0.012 | 0.007 |  |
| **4-HDoHE** | **DHA** | 0.0001 | 0.209 | 0.157 | 0.113 | 0.059 |  |
| 4k DHA | DHA | 0.9824 | 0.003 | 0.003 | 0.003 | 0.003 |  |
| 7-HDoHE | DHA | 0.7131 | 0.01 | 0.011 | 0.003 | 0.002 |  |
| 8-HDoHE | DHA | 0.2117 | 0.034 | 0.039 | 0.009 | 0.006 |  |
| **CYP Pathway** | |  |  |  |  |  |  |
| 12,13 EpODE | ALA | 0.9971 | 0 | 0 | 0 | 0 |  |
| 18-HEPE | EPA | 0.6786 | 0.01 | 0.02 | 0.002 | 0.002 |  |
| 16,17 EpDPE | DHA | 0.9677 | 0.003 | 0.002 | 0.002 | 0.001 |  |
| 19,20 DiHDoPE | DHA | 0.9996 | 0.001 | 0 | 0.001 | 0 |  |
| 20-HDoHE | DHA | 0.9971 | 0.014 | 0.018 | 0.004 | 0.002 |  |
| **ω-6 FA** | |  |  |  |  |  |  |
| **LOX Pathway** | |  |  |  |  |  |  |
| 9,10,13 triHOME & 9,12,13 triHOME | LA | 0.9708 | 0.012 | 0.01 | 0.013 | 0.006 |  |
| **9-HODE** | **LA** | <0.000001 | 0.351 | 0.489 | 0.091 | 0.065 |  |
| **13-HODE** | **LA** | <0.000001 | 0.212 | 0.324 | 0.057 | 0.037 |  |
| 15-HETrE | DGLA | 0.5769 | 0.015 | 0.022 | 0.004 | 0.004 |  |
| 8-HETrE | DGLA | 0.6841 | 0.011 | 0.015 | 0.003 | 0.003 |  |
| 13-HOTrE-y | GLA | 0.9086 | 0.003 | 0.004 | 0.001 | 0.001 |  |
| 11-HETE | AA | 0.5769 | 0.028 | 0.034 | 0.008 | 0.005 |  |
| 12-HETE | AA | 0.6841 | 0.056 | 0.063 | 0.014 | 0.008 |  |
| 12-oxoETE | AA | 0.9086 | 0.018 | 0.014 | 0.015 | 0.01 |  |
| 15-HETE | AA | 0.0653 | 0.051 | 0.074 | 0.013 | 0.008 |  |
| 15-oxoETE | AA | 0.8512 | 0.006 | 0.005 | 0.002 | 0.001 |  |
| 5,15 diHETE | AA | 0.8224 | 0.009 | 0.008 | 0.004 | 0.003 |  |
| 5,6 diHETE | AA | 0.8716 | 0.005 | 0.005 | 0.002 | 0.002 |  |
| **5-HETE** | **AA** | <0.000001 | 0.42 | 0.322 | 0.219 | 0.111 |  |
| 5-oxoETE | AA | 0.6829 | 0.017 | 0.016 | 0.009 | 0.009 |  |
| 6t, 12epi LTB4 | AA | 0.5748 | 0.02 | 0.018 | 0.009 | 0.006 |  |
| 8,15 diHETE | AA | 0.0261 | 0.066 | 0.071 | 0.02 | 0.016 |  |
| 8-HETE | AA | 0.1113 | 0.043 | 0.054 | 0.011 | 0.007 |  |
| 9-HETE | AA | 0.0818 | 0.047 | 0.059 | 0.011 | 0.008 |  |
| tetranor 12-HETE | AA | 0.9895 | 0.001 | 0 | 0 | 0 |  |
| **COX Pathway** | |  |  |  |  |  |  |
| 12-HHTrE | AA | 0.889 | 0.004 | 0.007 | 0.001 | 0.001 |  |
| PGE2 | AA | 0.9902 | 0.001 | 0.001 | 0.001 | 0 |  |
| TXB2 | AA | 0.9786 | 0.001 | 0.002 | 0.001 | 0 |  |
| **CYP Pathway** | |  |  |  |  |  |  |
| 12,13 diHOME | LA | 0.98 | 0.002 | 0.002 | 0.001 | 0.001 |  |
| 12,13 EpOME | LA | 0.9758 | 0.002 | 0.001 | 0.001 | 0 |  |
| 9,10 diHOME | LA | 0.9935 | 0.001 | 0.001 | 0.001 | 0 |  |
| 11,12 DiHETrE | AA | 0.9989 | 0 | 0 | 0 | 0 |  |
| 11,12 EpETrE | AA | 0.9992 | 0 | 0 | 0 | 0 |  |
| 14,15 DiHETrE | AA | 0.9993 | 0 | 0 | 0 | 0 |  |
| 14,15 EpETrE | AA | 0.9936 | 0.001 | 0 | 0.001 | 0 |  |
| 5,6 DiHETrE | AA | 0.9912 | 0.001 | 0 | 0.001 | 0 |  |
| 5,6 EpETrE | AA | 0.9987 | 0 | 0 | 0 | 0 |  |
| 8,9 DiHETrE | AA | 0.9987 | 0 | 0 | 0 | 0 |  |
| 5-iso PGF2aVI | AA | 0.9796 | 0.001 | 0.001 | 0 | 0 |  |
| 16-HETE | AA | 0.9926 | 0.001 | 0 | 0 | 0 |  |
| 20cooh AA | AA | 0.9945 | 0.008 | 0.005 | 0.008 | 0.002 |  |

**Table S6: Characteristics of ACPA+ and ACPA+ Progressors**

|  | ACPA+ (n = 27) | ACPA+ Progressor (n = 4) |
| --- | --- | --- |
| Age, mean (SD) | 50.0 (11.0) | 31.5 (1.9) |
| Female Sex (%) | 74.4% | 50.0% |
| Follow up (months), median (IQR) | 37.0 (80.5) | 77.5 (37.5) |
| BMI, mean (SD) | 29.8 (6.3) | 29.2 (10.9) |

**Figure S1: (A)** Scatter plot showing the total FA levels quantified in samples segregated based on the year of sample collection and the Spearman rank correlation with years of storage. **(B)** Scatter plot showing the total oxylipins in samples segregated based on the year of sample collection and the Spearman rank correlation with years of storage. (C) Scatter plot showing the concentrations of total FA mass and total oxylipin mass in all individuals segregated based on (+/-) NSAID use. Data analyzed by Mann-Whitney U test. Samples used for this analysis were collected between 2007-2017. (D) Scatter plot showing the concentrations of total FA mass and total oxylipin mass in all individuals segregated based on enzymatic pathway and (+/-) NSAID use. Data analyzed by Mann-Whitney U test. Samples used for this analysis were collected after 2013.

**Figure S2:** **Analysis and distribution of FAs in FDR.** Samples used for this analysis were collected in/after 2013. Box-Whiskers plots showing the % distribution of serum **(A)** MUFA and PUFA **(B)** SFA and UFA and **(C)** ω3, ω6, ω9, ω7 and ω5 FA subtypes in ACPA- FDR, and ACPA+ FDR. **** = P<0.0001, ns = not significant; data analyzed by Mann-Whitney U test.

**Figure S3:** Levels of serum oxylipins in ACPA+, ACPA- and ACPA+ Progressors after adjustment for sample storage time.

**
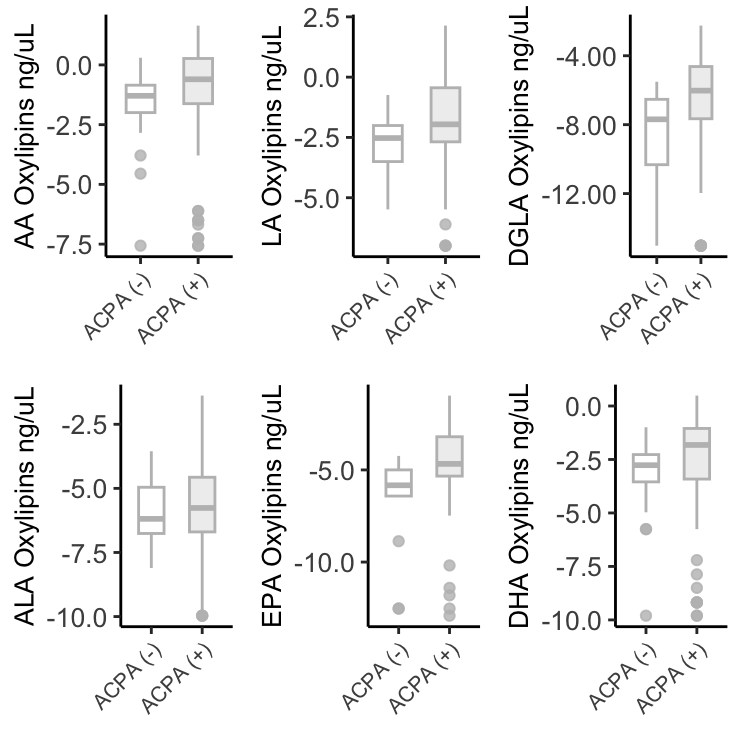
**

**
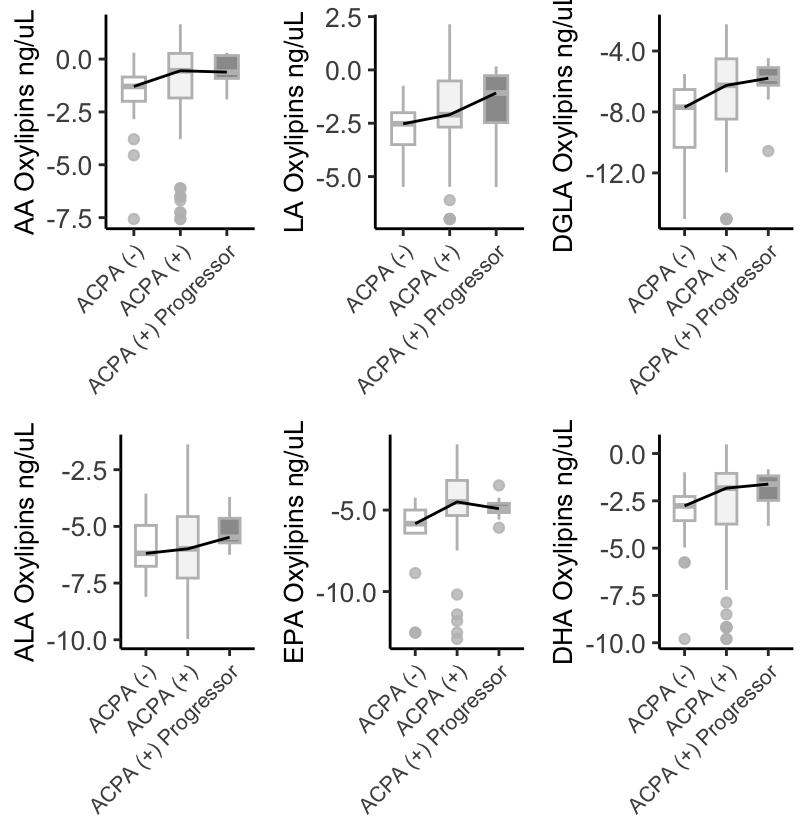
**

**
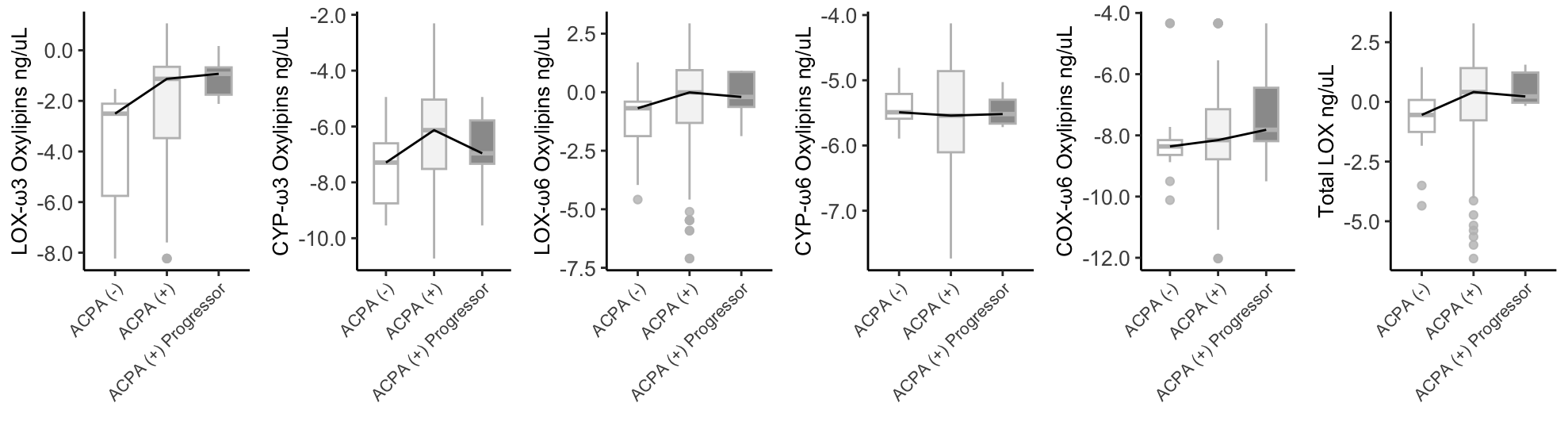
**

**Figure S4:** Box-Whiskers plot showing levels of ω3 and ω6 oxylipins in ACPA- FDR (N=10) and ACPA+ FDR (n=31). ** = P<0.01, ns = not significant. Data was analyzed using Mann-Whitney U test.

**Figure S5:** Consensus clustering of oxylipins revealed 8 distinct clusters, 2 of which were higher in ACPA+ FDR samples compared to ACPA-. Analyzed by Wilcoxon rank sum test. MDS: Multi-dimensional scaling.

**Figure S6:** Total Oxylipin levels in ACPA- FDR, ACPA+ FDR, and ACPA+ Progressors (top). Levels of AA, LA, DGLA, ALA, EPA and DHA derived oxylipins split by group.


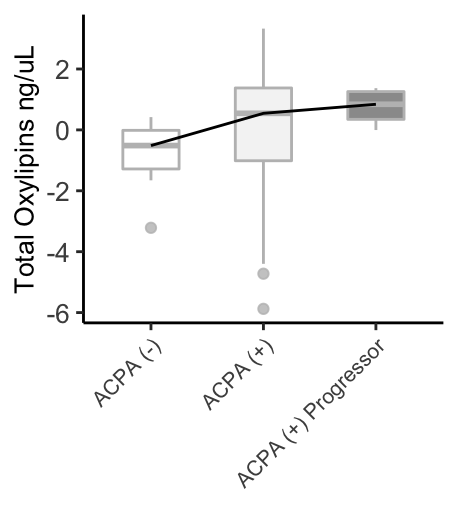


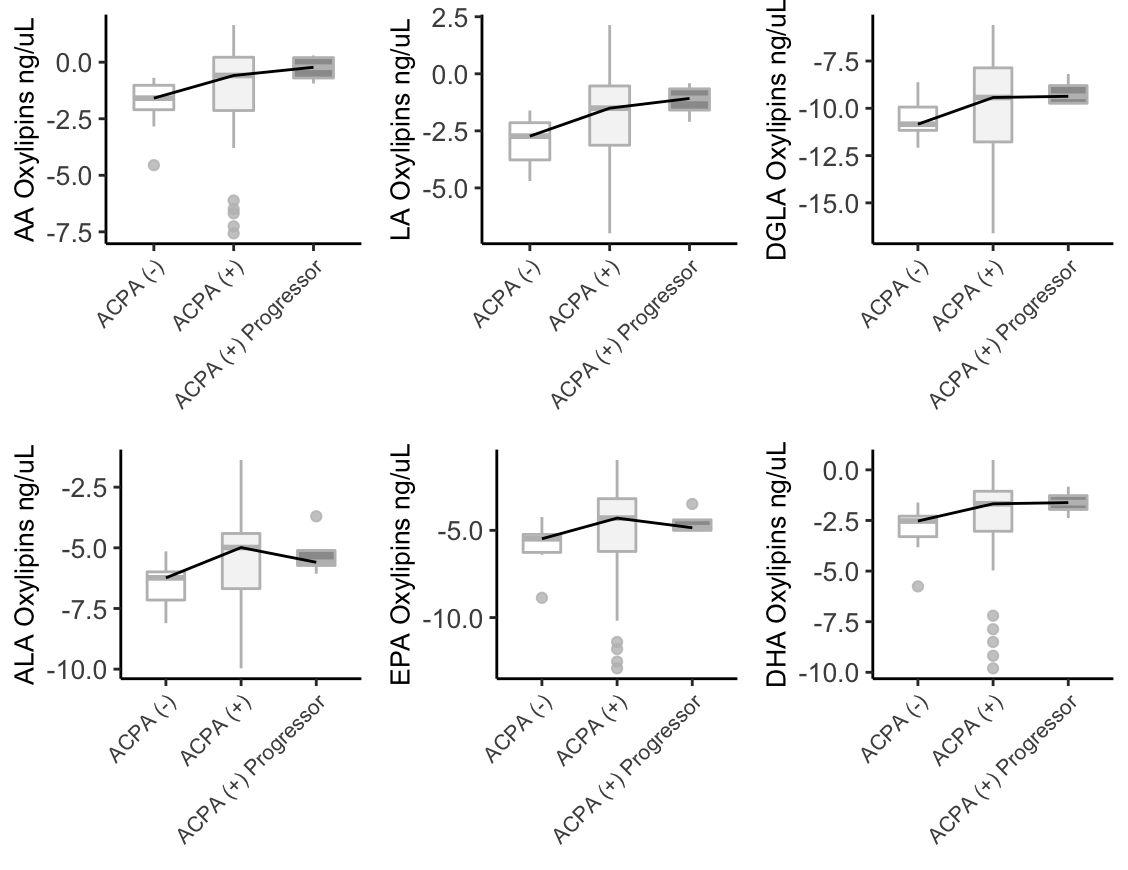

Supplement: Supplementary file 1 — Additional file1: Table S1. Effect of storage time on the levels of individual FAs – Columns highlighted in bold represent FAs that showed significant differential expression. Data was analyzed by student t test (assuming unequal variance) and q<0.05 was considered significant. Benjamini-Hochberg method was used to correct of false discovery rate (represented as q-values). Table S2. Clinical features of ACPA− and ACPA+ FDR. RF=rheumatoid factor; CRP = C-reactive protein; DAS28 = disease activity score 28; anti-CCP = anti-cyclic citrullinated protein antibodies; BMI = Body Mass Index. #Pearson Chi-square test; $Mann-Whitney U test; statistically significant values are indicated in bold. ‘-‘ indicates absence of any value. Table S3. Characteristics of ACPA+ samples selected for fatty acid and oxylipin analysis, categorized by either i. sample acquired at inception study visit or ii. sample acquired after longitudinal follow-up. Table S4. Table showing differences in FA levels between ACPA+ and ACPA− FDR. Data analyzed Mann-Whitney U test and false-discovery rate was corrected using Benjamini-Hochberg method. Significant values are indicated in bold. Table S5. Table showing differences in individual oxylipin levels between ACPA+ and ACPA− FDR. Data is represented as mean ± SD. P values were obtained after performing Student t-test and correcting for multiple comparisons using Bonferroni-Dunn method. Significant values were indicated in bold. Table S6. Characteristics of ACPA+ and ACPA+ Progressors. Figure S1. (A) Scatter plot showing the total FA levels quantified in samples segregated based on the year of sample collection and the Spearman rank correlation with years of storage. (B) Scatter plot showing the total oxylipins in samples segregated based on the year of sample collection and the Spearman rank correlation with years of storage. (C) Scatter plot showing the concentrations of total FA mass and total oxylipin mass in all individuals segregated based on (+/-) N [file 13075_2024_3274_MOESM1_ESM.docx]
